# Innovations in Gerontology Education: A Multifaceted Approach

**DOI:** 10.1093/geroni/igaa057.1779

**Published:** 2020-12-16

**Authors:** Melissa O’Connor, Melissa O’Connor

## Abstract

By 2030, older adults are projected to outnumber children. This growth among older adults presents both opportunities and challenges on how to care for this unprecedented older adult population. In 2010, there were 7.2 family caregivers for every person 80 and older; this is expected to fall to 3 by 2050. Many older adults are at risk of becoming an elder orphan - someone aging alone with no family available to address their caregiving needs. This underscores the urgent need for the education of nurses who are prepared to care for older adults with complex social and health needs, many of whom are suffering from three to five chronic conditions. This symposium highlights cutting-edge research designed to transform nursing education to better prepare students to advance the health of older adults. Our first presentation describes ageism among undergraduate students and innovative curricula to positively influence their preference for working with older adults. Next, the groundbreaking tool-kit that encourages self-reflection on beliefs about aging and reframes how students view older adults. Third, the qualitative results of a collaborative learning experience designed to introduce students earlier and more often to older adults. Finally, findings from a student-led study investigating the unique needs of caregivers of older adults with diabetes highlights the importance of funding students to conduct research related to older adults. Our symposium ends with a discussion of how these approaches are transforming nursing education to adequately prepare nurses to improve the health and health care of older adults.

